# 磁性离子印迹技术用于元素形态分离分析

**DOI:** 10.3724/SP.J.1123.2022.07013

**Published:** 2022-11-08

**Authors:** Yifan PAN, Feng ZHANG, Wei GAO, Yuelun SUN, Sen ZHANG, Hongzhen LIAN, Li MAO

**Affiliations:** 1.生命分析化学国家重点实验室, 南京大学化学化工学院, 南京大学现代分析中心, 江苏 南京 210023; 1. State Key Laboratory of Analytical Chemistry for Life Science, School of Chemistry & Chemical Engineering, Center of Materials Analysis, Nanjing University, Nanjing 210023, China; 2.现代毒理学教育部重点实验室, 南京医科大学公共卫生学院, 江苏 南京 211166; 2. Ministry of Education (MOE) Key Laboratory of Modern Toxicology, School of Public Health, Nanjing Medical University, Nanjing 211166, China

**Keywords:** 离子印迹技术, 元素形态, 固相萃取, 分离, 富集, 综述, ion imprinting technology, elemental speciation, solid-phase extraction, separation, enrichment, review

## Abstract

元素的形态决定了其在环境和生物过程中的不同行为,形态分析正在被分析化学、环境化学、地球化学、生态学、农学和生物医学等众多学科所关注。环境和生物样品基质复杂、化学形态多样、含量低且易转化是元素形态分析面临的挑战,因此对元素形态的甄别、定量、生态毒性评价和生理功能研究需要对原生形态进行高选择性识别和高效率分离。固相萃取是一种有效应对以上难题的方法,但现有材料和方法远不能满足要求。离子印迹聚合物可与印迹金属离子特异性结合,具有准确、灵敏、可靠的特点,近年来在元素形态分离富集和分析检测方面得到了较为广泛的应用。鉴于非磁性吸附剂在固相萃取操作时,需要将分散在样品溶液中的吸附材料经过离心或过滤分离,操作比较繁琐费时,而磁性材料易被外部磁场快速分离,因此操作简便快速的磁固相萃取正成为元素形态分离富集中一种极具潜力的方法。这篇综述系统总结了离子印迹技术的最新进展,包括离子印迹技术的原理、离子印迹聚合物的制备方法,并根据元素形态分析中离子印迹磁固相萃取的发展现状,分析了离子印迹技术所面临的挑战,最后对元素形态分析中离子印迹技术的未来发展方向和策略提出了建议,提出开发基于有机-无机杂化聚合的多功能磁性离子印迹纳米复合物用于样品的前处理是建立识别选择性高、分离能力强、吸附容量大、形态稳定性好的形态分析方法的一种重要举措。

## Abstract (en)

Metal and metalloid elements have various possible isotopic compositions and oxidation states and often form coordination or covalent compounds with inorganic and organic small molecules or biological macromolecules, resulting in complex elemental speciation. Different species of the same element often have different properties, which dictate their behavior. Thus, elemental speciation analysis is vital for comprehensively and accurately assessing an element’s environmental and biological effects and the corresponding risks. Because elemental speciation determines the behavior of an element in different environmental and biological processes, the analysis of elemental species has, in recent years, been important in various subjects, including analytical chemistry, environmental chemistry, geochemistry, ecology, agronomy, and biomedicine. The complexity of environmental and biological sample matrices, as well as the multiformity, low levels, and lability of chemical forms pose severe challenges in elemental speciation analysis. Therefore, the highly selective identification and efficient separation of native species is necessary for conducting the identification, quantification, ecotoxicity evaluation, and physiological function study of elemental speciation. Sample pretreatment by solid-phase extraction is an effective solution to the aforementioned problems, but the existing methods do not meet the requirements of current research. The transition of the target species from pre-processing to the detection device includes both on- and off-line arrangements. Compared with the on-line approach, the off-line approach requires more manual participation, increasing the analysis workload. However, the off-line approach can improve the analysis efficiency through high-throughput pretreatment when large batches of samples are encountered, meaning the off-line approach is still an effective model. Ion imprinting technology has been developed based on existing molecular imprinting technology, with four main steps present in the synthesis of ion imprinted polymers. First, ion imprinting technology uses metal ions as templates. Then, these templates are combined with the functional monomers through coordination, electrostatic or hydrogen bonding. The functional monomers simultaneously surround and fix the templates, after which the cross-linkers and functional monomers polymerize to prepare ion-imprinted polymers with a specific structure and composition. Finally, the imprinted holes are created in the polymers by eluting the template ions. Therefore, the template molecules, functional monomers, and cross-linkers are three precursors necessary for synthesizing ion-imprinted polymers. These polymers can specifically bind to the imprinted metal ions with accuracy, sensitivity, and reliability. In recent years, they have been widely used in separating, enriching, analyzing, and detecting elemental species. During solid-phase extraction, the non-magnetic adsorbent materials dispersed in the sample solution need to be separated by centrifugation or filtration, which is time-consuming and laborious. Because an external magnetic field can be used for rapid magnetic solid-phase extraction, it has become a potential method for separating and enriching elemental species.

This review systematically summarizes the latest progress in ion-imprinting technology, including its principle and the preparation methods of ion-imprinted polymers. The challenges faced by ion imprinting technology are analyzed in the context of the development of ion-imprinting magnetic solid-phase extraction in elemental speciation analysis. Finally, the direction of future development and the strategies of ion imprinting technology in elemental speciation analysis are proposed. It is important to exploit novel organic-inorganic hybrid polymerization-based multifunctional ion-imprinted magnetic nanocomposites for the magnetic solid-phase extraction and separation of elemental species. By establishing the pretreatment protocols with high recognition selectivity, strong separation ability, large adsorption capacity, and good speciation stability, we expect to achieve the research objectives of simultaneously separating and enriching the multiple-species of typical metal/metalloid elements in environmental and biological samples.

金属及类金属元素不但具有不同的同位素组成和氧化态,而且常常与无机及有机小分子、生物大分子形成配位化合物或共价化合物(如金属有机化合物)等,元素的形态决定了其在环境和生物过程中所表现出来的不同行为,例如元素在环境和生物体系中的价态、聚集方式、结合分子结构及性质等决定了其生态效应、生物功能和毒性作用等^[1]^;元素在土壤、沉积物和地表水中的归趋主要取决于其与有机质、氧化物和泥土等的结合^[2]^;大气颗粒物中的元素形态决定着它们的生物有效性和毒性^[3]^。因此,元素形态分析对于全面正确地揭示元素的环境和生物影响及行为、获得其有关风险性的信息具有非常重要的意义^[2]^。

由于原子光谱和元素质谱在现阶段只能测定元素的总含量,尚不能区分其形态,所以形态分析一般采用先分离后分析的策略。分离-分析在线联用目前是形态分析的主流技术,常用的分离手段有高效液相色谱(HPLC)^[4⇓⇓-7]^、毛细管电泳(CE)^[4,8]^和气相色谱(GC)^[9]^等,测定手段则主要有石墨炉原子吸收光谱(GF-AAS)、氢化物发生-原子吸收光谱(HG-AAS)、原子荧光光谱(AFS)和电感耦合等离子质谱(ICP-MS)^[10]^等。其中,ICP-MS能够同时测定多种元素,并可提供同位素丰度比,具有较高的灵敏度,而且线性范围较宽,在所有的用于元素形态分离分析的联用技术中以HPLC-ICP-MS技术最为成熟。

然而,上述分离-分析联用技术离环境和生物样品中元素形态分析的要求还有相当大的距离^[11]^,这是因为:①用于形态分离的色谱柱的种类还很少,不能满足多元素、多形态的分离需求;②样本中待测单个形态的含量很低,分析的可检出性受到影响;③ HPLC、CE和GC分离一般持续数分钟甚至数十分钟,而且需要较高的柱压、电压或柱温,在分离的同时一些不稳定的形态会发生变化。因此,要实现元素形态的精准分析,需要在检测之前(包括在用联用技术分析之前)实施无破坏性的样品前处理^[12,13]^,以实现如下目标:①分离富集痕量目标组分(提高灵敏度); ②消除或减少样品基体(降低干扰); ③转移目标分析物至兼容后续检测的介质中(转换基体)。传统的形态样品前处理技术有液-液萃取(LLE)包括液相微萃取(LPME)等^[14]^,近年来更受重视的则是基于固相吸附材料的萃取技术,包括固相萃取(SPE)、基质固相分散(MSPD)、固相微萃取(SPME)等^[15⇓-17]^。目标形态从前处理过渡到检测装置包括在线和离线两种模式。相对于在线联用,离线联用需要较多的人工参与,增加了分析工作量,但是离线联用在面对大批量样品时,可以通过高通量的前处理提高分析效率,在现阶段仍不失为一种有效的模式。

## 1 元素形态的固相萃取分离富集

作为一类有效的分离富集方法,SPE是环境和生物分析化学中最常用的样品前处理手段之一^[18]^。先将气相或液相介质中的目标分析物通过吸附、静电、氢键、离子交换、配位、疏水、亲和或其他物理、化学或生物作用保留在固相吸附剂上,然后用合适的洗脱剂使目标组分与固相材料分离。相比传统的LLE, SPE因具有简便、快速、经济、环保的优点,被越来越多地应用于元素形态分析的样品前处理中。纳米材料则由于比表面积大、吸附容量大、承载作用位点丰富而备受重视。近年来,已经有许多功能化纳米材料被用于元素形态的SPE分离富集,如碳纳米管(CNTs)、石墨烯、金属有机框架(MOFs)^[19]^、金属氧化物和非金属氧化物等^[16,17,20]^,最近也出现了使用聚合物膜^[21]^和高分子纤维^[22]^作为元素形态SPE吸附剂的报道。但是,非磁性吸附剂在SPE操作时要将吸附材料分散在样品溶液中,这样的MSPD过程需要经过繁琐的离心或过滤分离,操作比较费时。

由于功能化磁性纳米复合物同时具有功能化材料对目标分子的选择性以及磁性材料易被外部磁场快速分离的能力,磁固相萃取(MSPE)在复杂环境和生物体系中痕量目标物分离富集方面发挥着积极作用,正成为元素形态分离富集中一种极具潜力的方法^[23]^。但也必须看到,作为一种SPE技术,大部分的磁性纳米材料对元素形态的分离还是基于某种修饰的功能基团,结合调节萃取条件如溶液pH来控制选择性,严格说来还是属于操作定义上的形态分级(fractionation)过程^[1]^。例如,巯基和氨基功能化的磁性纳米粒子(MNPs)分别通过配位作用和静电作用对三价砷(As(Ⅲ))和五价砷(As(Ⅴ))产生保留^[24]^,但所修饰的巯基和氨基对其他金属离子也会有不同程度的作用,也就不可避免带来一定的干扰,影响形态分析的结果。

## 2 离子印迹技术

分子印迹技术(MIT)是一种人工构建的、在空间构型和作用位点方面与特定的目标分子相匹配的一种技术。利用该技术(见图1)制备的分子印迹聚合物(MIPs)可特异性地识别特定的目标分子^[25]^。MIT具有如下特点:①预制性。根据所需将选择的模板分子和功能单体反应生成具有与预期相同印迹腔的MIPs。 ②识别选择性。除去印迹材料的模板分子后,聚合物中留下与目标分子的大小、形状、官能团和空间立体几何互补的结合位点和空间结构。③实用性。操作步骤简单,可适用不同酸碱性及温度等应用环境,已广泛应用于多个领域,如工业、催化、医学、传感器和色谱分析等。

**图1:**
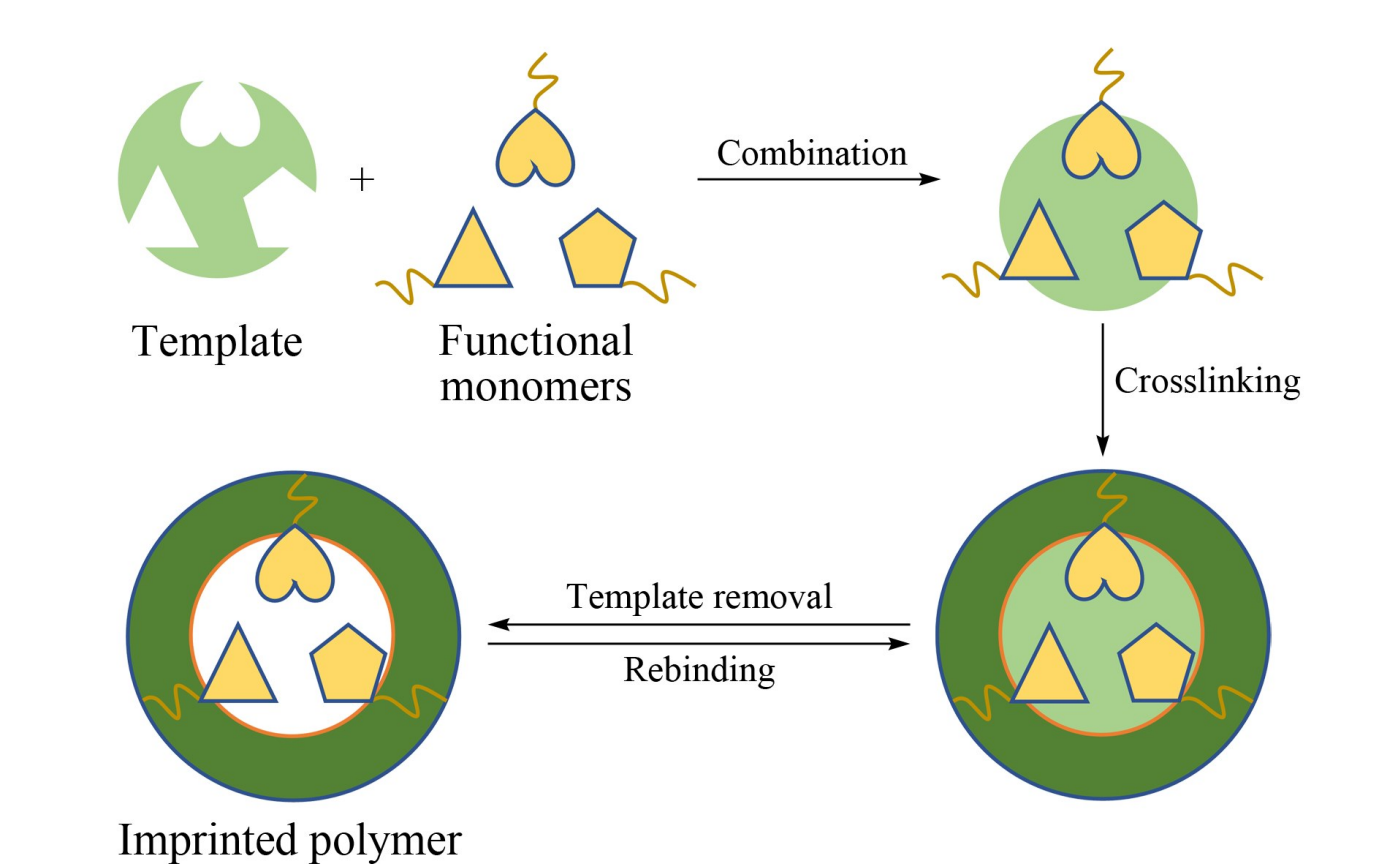
分子印迹聚合物合成示意图

离子印迹技术(IIT)在MIT的基础上发展而来,是MIT的一个重要发展方向^[26]^。IIT以金属离子为目标离子,通过其模板与功能单体发生配位、静电或氢键等作用结合,功能单体包围并固定模板,然后经交联聚合制备出具有特定结构和组成的离子印迹聚合物(IIPs),最后再通过洗脱去除目标离子,聚合物中留下具有特异性识别位点的印迹孔穴,可以对目标离子产生选择性识别^[27]^。

如图2所示,在IIPs制备过程中,除了常规的目标离子-功能单体-交联剂直接聚合(Route 1),经常需要一种能与目标离子发生相互作用(如配位、静电或氢键)的配体形成复合印迹模板,再与功能单体和交联剂聚合(Route 2),一方面提高印迹选择性,另一方面维持目标形态离子在反应过程中的稳定性。如果配体本身同时也能通过交联剂聚合,则在反应中可以将该配体用作功能单体(Route 3)。

**图2:**
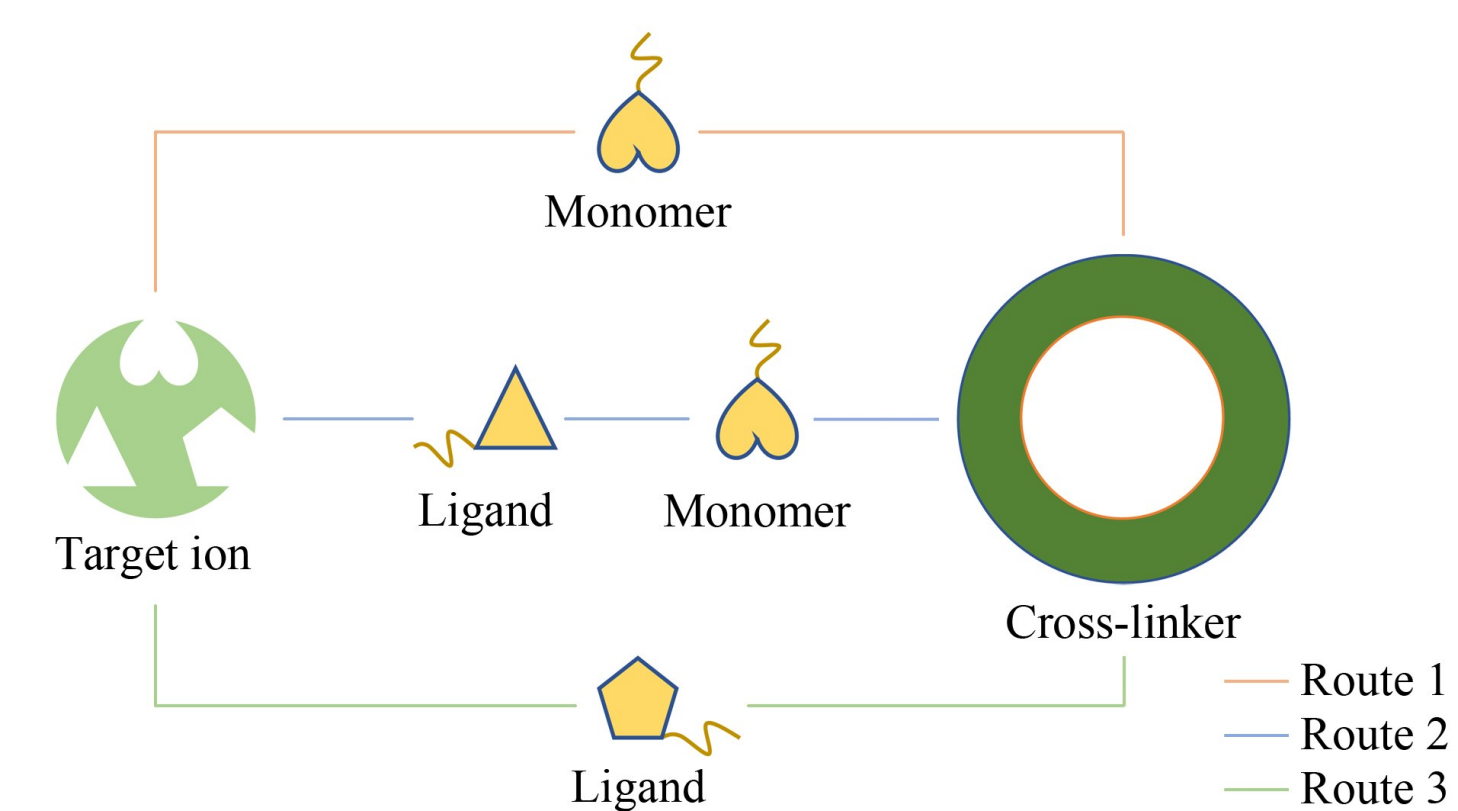
离子印迹聚合物制备过程中反应物的3种组合方式

由于对离子的特异性识别和选择性富集,IIPs已经被应用于元素的形态分析^[16,20,26]^。基于目标离子与配体之间不同的作用力,印迹聚合物通常有两种典型的合成方法,分别是预组装法和自组装法^[16]^。预组装法将两种组分以共价键结合,通过此方式合成的印迹聚合物结构稳定,选择性较高,但材料的吸附及洗脱过程较为缓慢,尤其对于不太稳定的形态离子应用受到一定的限制;自组装法通过两种组分之间的配位、静电或氢键等相对较弱的作用力,经此方法合成的印迹材料可方便且快速地完成吸附与洗脱。目前,自组装法已被越来越多地运用于金属离子特别是元素形态的分离富集。

### 2.1 离子印迹聚合物的聚合前体

离子印迹聚合物中可供选择的功能单体种类非常丰富,使用频率较高的功能单体是甲基丙烯酸(MAA)、丙烯酸(AA)、4-乙烯基吡啶(4-VP)、2-乙烯基吡啶(2-VP)、1-乙烯基咪唑(1-VI)和丙烯酰胺(AM)等。一些用于溶胶-凝胶(Sol-gel)反应的含乙烯基的共聚前体如3-(甲基丙烯酰氧)丙基三甲氧基硅烷(*γ*-MAPS)和乙烯基三甲氧基硅烷(VTMOS)等也可以用作功能单体。但普适性的功能单体大多能与多种金属离子发生作用,因而往往采用双功能单体组合的方式来提高印迹选择性。除了用以上这些功能单体组合外,最常用来作为第二功能单体的还有甲基丙烯酸-2-羟基乙基酯(HEMA)^[28⇓⇓-31]^等,近来也有3种功能单体如1-VI/烯丙基硫脲(ATA)/吡啶-3-羧酸(ANA)^[32]^、对氨基苯甲酸(PABA)/间苯二酚/乙二醛^[33]^组合的报道。

对于易变形态的目标离子,大多是在印迹反应前采用选择性的配体与之形成复合模板,如利用巯基苯并咪唑(MBI)或巯基苯并噻唑(MBT)与甲基汞(Hg(Me)^+^)配位^[34]^,更方便的是采用本身兼具配体作用的功能单体,如用MAA^[35]^、1-VI^[36]^或ATA^[37]^与Pb^2+^配位,用乙烯基磷酸二甲酯(DMVP)与Sn^2+^配位^[38]^,用组氨酸(His)和甲基丙烯酰氯(MAC)反应生成的甲基丙烯酰氨基组氨酸(MAH)与Cr^3+^配位^[39]^,用3-乙烯基苯甲醛(3-EB)经*N*-月桂基-2-溴噻唑催化缩合生成的间乙烯基安息香(*m*-VB)与有机锡化合物(OTCs)配位^[40]^。4-VP则通过其氨基质子化与Cr_2_O_7_^2-^发生静电作用以提高印迹选择性,并维持Cr(Ⅵ)形态的稳定性^[28,29]^。与采用双功能单体的效果类似,为了提高IIPs的选择性,许多时候也预先采用双配体组合,如用MAA和4-VP组合与Pb^2+[41]^或As(Ⅲ)^[42]^形成配合物。一些自带配体的共聚前体也可以用来作为配体与形态离子构建复合模板,如利用3-氨基丙基三乙氧基硅烷(APTES)的氨基与Cr^3+^之间的配位作用^[43]^、利用3-(2-氨基乙基氨基)丙基三乙氧基硅烷(AEAPTES)的氨基与Cu^2+^之间的配位作用^[44]^、利用3-(2-氨基乙基氨基)丙基三甲氧基硅烷(AEAPTMS)分子上质子化的氨基与Cr(Ⅵ)^[31]^或As(Ⅴ)^[45]^阴离子之间的静电作用。另一方面,高度特异性的配体一直都引人注目。Chen课题组和Guan课题组合作^[46]^,先由胸腺嘧啶(T)和碳酸乙烯酯(EC)反应生成*N*-1-(2-羟乙基)胸腺嘧啶(T-OH),再与3-异氰酸基丙基三乙氧基硅烷(IPTS)反应生成连接胸腺嘧啶的T-IPTS,以T-IPTS与Hg^2+^形成的配合物(IPTS-T-Hg^2+^-T-IPTS)与四乙氧基硅烷(TEOS)经过Sol-gel反应制备得到的Hg^2+^-IIP,展现了非常高的选择性。

目前,常用的交联剂有二甲基丙烯酸乙二醇酯(EGDMA)、三羟甲基丙烷三甲基丙烯酸酯(TMPTM)、二乙烯基苯(DVB)和环氧氯丙烷(ECH)等,引发剂则有偶氮二异丁腈(AIBN)、过氧化苯甲酰(BPO)和过硫酸铵(KPS)等。在IIPs的制备过程中,有时需要加入乙腈、乙醇、甲苯或氯仿等作为致孔剂,也可以通过加入表面活性剂如十六烷基三甲基溴化铵(CTAB)胶束^[29]^或纳米金属氧化物如纳米ZnO^[47]^作为第二模板,在形成目标离子孔穴的同时另外形成一些不同尺寸的孔穴,以增加印迹材料的通透性和目标离子传输的速度。

### 2.2 离子印迹聚合物的制备方法

印迹聚合物的基本合成技术包括基于分步聚合机理的Sol-gel反应和基于链聚合机理的自由基聚合(FRP)反应^[26,27]^。印迹聚合物的制备方法有本体聚合法、沉淀聚合法、悬浮聚合法、乳液聚合法和表面印迹法等。本体聚合是传统的合成方法,其制备操作简单,工艺成熟,应用较为广泛。将模板、功能单体、交联剂和引发剂按照一定的比例溶解于溶剂中,在特定条件下制得印迹聚合物,经干燥、粉碎、筛分等后处理,最后使用合适的洗脱剂洗掉目标离子。然而,用该方法合成的材料后续处理比较繁琐,研磨过程可能会破坏印迹材料的部分识别位点及形貌。我们课题组^[44]^结合“一锅法”Sol-gel共缩合,以AEAPTES为功能单体,Cu^2+^为模板,TEOS为交联剂,在石英毛细管中制备了一种新型氨基功能化的Cu^2+^离子印迹有机-无机杂化整体柱(Cu^2+^-IIHMC)用于Cu^2+^的选择性分离。这个工作采用CTAB作为第二模板/致孔剂,得到了空穴丰富、通透性好的离子印迹整体材料,实际上是一种改进的本体聚合法。沉淀聚合也常被用来制备印迹聚合物,其方法与本体聚合法相类似,但需要将印迹组分分散到大量的溶剂中,通过加热或光照引发聚合,生成粒径大小均一的不溶于溶剂的微球。此法不需要后续处理,但欲用该方法获得粒径均一的聚合物颗粒对所选溶剂的黏性有一定的要求;悬浮聚合将参与反应的各组分置于有机相中,通过快速旋转搅拌,升高体系温度引发聚合,有机相形成多个悬浮的小液滴,在液滴内发生聚合反应。该方法一般用于制备微米级印迹微球颗粒,但合成过程繁琐,对有机溶剂的要求较高;乳液聚合与悬浮聚合类似,也可制备出颗粒较为均匀的印迹微球,但反应需要有乳化剂参与,反应过程较复杂,且乳化剂不易清除干净。

### 2.3 表面离子印迹聚合物

表面印迹技术是近年来颇为常用的一种IIPs制备方法,它是在固相基质表面进行聚合反应使得目标离子的识别位点均匀地分散在基质表面上的一种技术。经该法制得的印迹材料易获得选择性结合位点且模板结合速率快,目标离子容易被洗脱,分离效率高,还可降低非特异性吸附,减少包埋现象的发生。表面印迹法又可分为化学接枝法、聚合加膜法、牺牲载体法等类型。常用的提供印迹表面的固相载体有硅胶、碳纳米管(CNTs)、氧化石墨烯(GO)等。Fan等^[45]^以AEAPTMS为配体和功能单体,ECH为交联剂,在作为固相载体的SiO_2_微球表面制备了一种As(Ⅴ)-IIP。Liu等^[43]^以APTES与Cr^3+^形成复合模板,和TEOS在介孔二氧化硅SBA-15表面通过Sol-gel过程制备了一种Cr^3+^-IIP。张与合作者^[48]^以壳聚糖(CTS)改性的多壁碳纳米管(MWCNTs)经VTMOS烯基化后作为基体材料,以经VTMOS烯基化的CTS为功能单体,EGDMA为交联剂,AIBN为引发剂,在MWCNTs表面制备了一种Pb^2+^-IIP。Mishra和Verma^[37]^以ATA兼作配体和功能单体,加入EGDMA和AIBN,合成了碳纳米纤维(CNF)表面接枝的Pb^2+^-IIPs。Ma课题组^[31]^用TEOS处理GO作为基体材料,将质子化的AEAPTMS通过静电作用与Cr(Ⅵ)形成复合模板,加入HEMA作为第二功能单体、CTAB为致孔剂,在EGDMA和AIBN存在下,制备了一种Cr(Ⅵ)-IIP。Ma等^[19]^以FeCl_3_为模板、聚吡咯(PPy)为功能单体、UiO-66为基体,在UiO-66笼中对具有阴离子交换行为的PPy原位聚合生成Cl-IIP@UiO-66,涂覆到钛网上获得了基于MOFs的导电氯离子印迹聚合物膜电极,用于电化学选择性分析Cl^-^。Xi等^[21]^开发了一种用于膜制造的分子水平设计方法,以Cd^2+^为模板、3-丙烯基洛丹宁(3-AR)为功能单体、EGDMA为交联剂、AIBN为引发剂,将离子印迹聚合物层修饰到聚偏氟乙烯(PVDF)膜上;在该离子印迹膜(IIM)中,沿着膜孔制造了特定的主-客体结合位点,IIM与Cd^2+^绑定,通过IIM渗透以便有选择地分离Cd^2+^与其他离子。Kong等^[22]^将2-(二甲基氨基)乙基甲基丙烯酸酯(DMAEMA)通过高电子束辐照接枝到聚丙烯(PP)纤维上,用ECH对生成的纤维(PP-g-DMAEMA)改性,以Cr(Ⅵ)为模板,1,6-己二胺(HA)为交联剂,结合高电子束辐照预接枝和表面离子印迹法合成了一种新型Cr(Ⅵ)-IIP。

## 3 面向元素形态分析的离子印迹新策略

### 3.1 有机-无机杂化材料

有机-无机杂化材料是一类新兴的功能材料,它集中了有机材料和无机材料各自的优点,功能基团的修饰丰富、灵活,材料机械强度高,热稳定性好,溶剂耐受性强,能适应较宽的pH范围,而且制备简单^[49]^。如果反应在石英毛细管中进行,通过加入致孔剂,制成的杂化整体柱可用于管内固相微萃取(in-tube SPME)或毛细管微萃取(CME)^[50]^。杂化材料功能基团的连接方式主要分为两类:一类是用含功能基团的硅烷和四烷氧基硅烷如TEOS作为前驱体,通过Sol-gel反应直接导入功能基团,或者用含活性基团的硅烷和四烷氧基硅烷如TEOS通过Sol-gel反应先引入乙烯基、氨基、巯基、环氧等活性基团,再通过这些活性基团进一步连接所需的功能基团;另一类是在乙烯基化杂化材料制备体系中加入含乙烯键的功能单体和自由基引发剂,这种方法被称为“一锅法”。在反应过程中,有机功能单体的共聚和烷氧基硅烷的缩合同时进行,通过FRP反应,实现功能基团的一步导入,而且亲水的和疏水的多种功能单体都可以参与反应。经“一锅法”制得的杂化材料功能基团分布更加均匀,密度更高,性能更加优越,为功能多样化且结构稳定的有机-无机杂化材料制备提供了新的方案。虽然基于杂化材料的IIPs已经开始被用作元素形态的SPE分离富集,但基本上都是只经过Sol-gel反应生成的杂化IIPs,如[SiO_2_@NH_2_-Cr^3+^-APTMS](APTMS: 3-氨基丙基三甲氧基硅烷)^[51]^、SBA-15@[TEOS-APTES-Cr^3+^]^[52]^和MGO@[TEOS-MPTMS-As(Ⅲ)]^[53]^以及通过反应形成固相的[TEOS-TPED-Hg^2+^](TPED: *N*-3-(三甲氧基硅基)丙基乙二胺)^[54]^和[TEOS-(IPTS-T-Hg^2+^-T-IPTS)]^[46]^。Sol-gel过程中的FRP可以提供更丰富的有机功能基团,从而为元素不同形态提供选择性更高的结合位点及其组合,但目前结合Sol-gel和FRP反应生成的IIPs只有极少的报道,如通过反应形成固相的[TEOS-*γ*-MAPS-(1-VI-Pb^2+^)]^[36]^,但这个工作只考察了该Pb^2+^-IIPs相对于Cu^2+^、Cd^2+^和Zn^2+^的选择性。

### 3.2 可逆加成断裂链转移聚合反应

相对于可控性较差的FRP反应,可逆加成断裂链转移(RAFT)聚合反应是实现可控/“活性”自由基聚合的主要方法之一。这种新型的聚合反应是在传统FRP体系中引入RAFT试剂,通过与自由基进行可逆加成/断裂反应实现分子链“活性”增长。可控/“活性”自由基聚合的特点在于它结合了自由基聚合和“活性”聚合的优点,一方面可以精确控制大分子链的增长,从而得到预设相对分子质量且相对分子质量分布窄的各种结构类型的聚合物(包括嵌段聚合物、规整结构的星形聚合物和梳状聚合物等),二是适用单体范围广、单体易共聚、聚合条件比较温和,并能应用于水相介质^[55]^。RAFT聚合也已经被引入到IIPs的制备中:Liu和合作者^[52]^利用苯基溴化镁(PMB)和二硫化碳(CS_2_)形成苯基双硫脂,再和溴化苄生成链转移试剂(CTA)苯基双硫脂。然后将CTA与预先经*γ*-MAPS烯基功能化的SiO_2_修饰GO、模板离子Ni^2+^、功能单体AM和交联剂EGDMA在引发剂AIBN存在下聚合生成Ni^2+^-IIP。他们^[56]^还将PMB和CS_2_生成的中间产物与预先苄基氯化的SBA-15反应生成SBA-15-RAFT试剂,再与模板离子Ce^3+^、功能单体4-VP、EGDMA和AIBN聚合生成Ce^3+^-IIP。后来他们^[57]^又制备了基于磁性SBA-15的*m*-SBA-15-RAFT试剂,与Ce^3+^、功能单体4-VP、交联剂*N*,*N*'-亚甲基双丙烯酰胺和AIBN聚合生成Ce^3+^-MIIP。但是,以上通过RAFT聚合反应制备的IIPs只是分别用其他几种金属阳离子考察了各自的相对选择性,并未涉及同一元素不同形态之间的相对选择性。

### 3.3 密度泛函理论模拟计算

“一锅法”会使制得的有机-无机杂化材料功能基团分布更均匀、密度更高且性能更加优越,但因为离子印迹杂化共聚物的合成涉及Sol-gel和FRP两个反应,这给反应原料配置的选择和优化带来了较大的实验工作量。模拟计算可以用来评估合成IIPs的组分以及它们之间的相互作用,假定形成的配合物在聚合后保留其化学结构,对预聚体系进行建模,同步考虑模板、配体、功能单体、交联剂和溶剂之间的相互作用,通过比较它们之间结合能的大小,可以减少选择IIPs合成组分或溶剂的时间和资源。密度泛函理论(DFT)方法通常用于计算聚合物组分选择的“结合能”。Mesa等^[34]^使用高斯09软件,采用M06-2X方法,对C、S、N、O和H原子采用6-31G(d)基组进行模拟,在Hg(Me)^+^存在的情况下,使用赝势(LANL2DZ)描述汞原子的核心轨道。通过构建金属离子-配体的复合物,用它的能量减去各自组成部分的能量,得到结合能,同样的过程也应用在金属离子-配体-功能单体的复合物上。通过理论计算和结合能的比较,他们发现在乙醇中Hg(Me)^+^和配体MBI、MBT的结合能最小,因此形成的复合模板最稳定。就功能单体而言,它对配体的结合能应高于与模板的结合能,以方便目标离子在IIPs中吸附和解吸。模拟计算结果表明,功能单体AA还能够通过氢键与MBI、MBT形成更加稳定的复合物。

### 3.4 离子印迹聚合物多功能化

目前,和应用于元素形态分析的常规SPE技术类似,基于IIT的形态分析一般有下列3种方案:①采用一种SPE材料,选择性分离某一种形态并定量,另一种形态含量通过从总量中差减得到。这样处理不能确定差减所得是否全部来自于另一种形态,常用的办法是将样品进行氧化/还原处理,经二次SPE后测定总量再差减。②采用一种SPE材料,在不同实验条件下(如溶液pH)分别萃取两种形态,或者在一个pH下萃取一种形态而在另一pH下同时萃取两种形态,再通过差减获得。③组合使用两种不同SPE材料,分别萃取特定的形态。以上这些方案除了操作繁琐、费时外,还存在形态定义界定不明确、容易对不稳定形态造成扰动、误差大或成本高等不足。近年来,在同一固相基质上集成多个功能基团以适应不同目标组分的分离富集已经形成一种趋势,用于元素及形态的多功能SPE材料虽然也开始有报道^[58⇓⇓-61]^,但总体上还非常少,可选性受到限制。

## 4 离子印迹技术在环境元素形态分析中面临的挑战

不难看出,IIPs开始在元素形态分析方面发挥作用,但还存在一些瓶颈问题亟待解决。①表面聚合已经成为IIT的主流技术,但目前大多采用带乙烯基的功能单体通过交联剂在引发剂存在下发生FRP反应,在固体支持物表面生成的聚合层是刚性较弱的有机聚合物,而且在溶剂中易于溶胀,这样会造成已生成的印迹腔不可恢复从而影响其性能^[26,27]^。而且,以SiO_2_、MWCNTs、GO、CNF以及Fe_3_O_4_作为固相支持物时,现有的IIPs往往需要多步反应合成,步骤较为复杂^[31,37,43,45,48]^。加上传统的FRP反应不易控制,难以按照预设制备所需结构且批间一致性好的IIPs。②尽管已有用不同阳离子作为混合模板离子印迹的报道,但真正意义上的对于同一元素的不同形态同时印迹的报道非常少见,而这一点对于形态分析具有特殊意义。Muñoz-Olivas课题组^[40]^合成了一种用于分离有机锡化合物(OTCs)的IIP,虽然能够将三丁基锡(Sn(Bu)_3_^+^)、二丁基锡(Sn(Bu)_2_^2+^)、一丁基锡(Sn(Bu)^3+^)和三苯基锡(Sn(Ph)_3_^+^)全部保留,一并经过洗脱后用GF-AAS测定这些OTCs的总量,但具体的Sn(Bu)_3_^+^、Sn(Bu)_2_^2+^、Sn(Bu)^3+^和Sn(Ph)_3_^+^仍需要通过GC进一步分离测定,而且无论是用Sn(Bu)^3+^还是Sn(Bu)_2_^2+^与*m*-VB形成的配位复合模板制备的IIP,对于不同的OTCs形态均没有选择性。Kisomi等^[38]^以DMVP为配体制备了一种Sn^2+^印迹聚乙烯基磷酸二甲酯纳米粉末(Sn^2+^-IPDMVPN),但没有提到对于OTCs的选择性。另外,虽然已经有一些As(Ⅲ)-IIPs和As(Ⅴ)-IIPs被合成,但用于一甲基砷(MMA)和二甲基砷(DMA)的IIPs尚未见报道。特别是有更多的Pb^2+^-IIPs被制备,但没有发现有关其他Pb形态如烷基铅化合物(ALCs)IIPs的工作。③目前我国大气颗粒物重金属浓度较高,其中As、Cd、Cr和Ni超标严重,部分地区Pb和Mn超标^[62]^,而重金属环境和健康的影响不仅取决于元素的种类和浓度,还取决于直接决定其生物可给性的价态、元素形态和化合物组成^[63]^。鉴于我国大气颗粒物污染的严峻现状,在继续开展水、土环境介质中元素形态分析的同时,亟须加大力度开展大气颗粒物中元素形态分析的方法学研究。④现有的IIPs在使用时,有时对目标离子的吸附pH有一定的限制。如基于功能单体4-VP和第二功能单体HEMA制备的Cr(Ⅵ)-IIP,合适的溶液pH为2~4^[28]^。以配合物间乙烯基安息香-钛酸四丁酯(*m*-VB-TBT)为复合模板制作的OTCs-IIP,其最佳印迹效果要在低而窄的pH值范围(2.5~3.5)才能展现^[40]^。然而,在这种pH条件下,Cr和Sn的原生形态可能已经发生了变化,而形态分析的一个关键点是要求维持易变形态在样品前处理时的稳定性。以上所述诸点可能是目前影响IIT用于元素不同形态分离分析的关键制约因素。

## 5 元素形态分析中离子印迹磁固相萃取现状

普通吸附剂分散在溶液中需要通过离心或过滤才能从体系中分离,而磁性吸附剂特有的磁响应特性使其在作为离子吸附剂时可以高效回收、使用方便、环境友好。磁固相萃取具有其他非磁性技术无法比拟的优势,因此在有害元素及形态吸附中受到越来越多的关注。

在磁性离子印迹聚合物(MIIPs)制备方面,近年来报道了一些主要以SiO_2_作为印迹聚合物(IP)中间媒介的具有Fe_3_O_4_@SiO_2_@IP结构的MIIPs。Taghizadeh和Hassanpour^[29]^用4-VP和Cr(Ⅵ)形成复合模板,然后和修饰纳米SiO_2_的磁性MWCNTs混合,以HEMA为第二功能单体、EGDMA和AIBN分别作为交联剂和引发剂,制备了一种Cr(Ⅵ)-MIIP。Liang等^[30]^以磁性GO(MGO)为固相载体,采用4-VP和HEMA为共同功能单体,在EGDMA和AIBN存在下,制备了另一种Cr(Ⅵ)-MIIP。Tavengwa等^[64]^用*γ*-MAPS在纳米Fe_3_O_4_表面修饰乙烯基,将4-VP与烯基功能化Fe_3_O_4_在BPO引发下生成聚4-VP修饰的Fe_3_O_4_纳米复合物,接着与1-溴丙烷进行季铵化反应生成磁性聚(*n*-丙基-4-乙烯基吡啶鎓),利用季铵盐与Cr(Ⅵ)离子之间的离子对作用生成Cr(Ⅵ)-MIIP。Cui等^[35]^用TEOS在纳米Fe_3_O_4_表面包裹SiO_2_,然后和*γ*-MAPS反应修饰乙烯基,以MAA-Pb^2+^配合物作为复合模板,加入EGDMA和AIBN,反应制得一种Pb^2+^-MIIP。Kong等^[32]^以经过*γ*-MAPS烯基化的Fe_3_O_4_@SiO_2_纳米颗粒为固相载体,以衣康酸和*N*-异丙基丙烯酰胺分别作为Pb^2+^的配位功能单体和辅助配位功能单体,以1-VI、ATA和ANA为共同功能单体,制备了另一种Pb^2+^-MIIP。Hu课题组^[47]^以经过*γ*-MAPS烯基化的Fe_3_O_4_@SiO_2_纳米颗粒为固相载体、4-VP作为Pb^2+^的配位功能单体、纳米ZnO作为牺牲模板,制备了一种吸附-解吸速度较快的Pb^2+^-MIIP。Ma课题组^[31]^以MGO为固相载体,采用3-巯基丙基三甲氧基硅烷(MPTMS)与TEOS在As(Ⅲ)存在下共聚,制备了一种As(Ⅲ)-MIIP。张课题组^[65]^合成了一种MGO和MOF的复合材料,以MGO/MIL-101(Cr)为固相载体、多巴胺为功能单体、Cu^2+^和Pb^2+^为混合模板离子,制备了一种针对这两种阳离子的MIIP。本课题组先用溶剂热法生成Fe_3_O_4_纳米颗粒,加入TEOS和*γ*-MAPS通过“一锅法”在Fe_3_O_4_纳米颗粒表面包裹烯基化的有机-无机杂化整体层,最后用1-VI和AM为功能单体,分别制备了Au^3+^-MIIP^[66]^和Ce^3+^-MIIP^[67]^。与传统的无机二氧化硅和有机聚合物整体材料相比,Sol-gel法制备的杂化整体材料具有比表面积大、机械/化学稳定性高、生物相容性好等优点,而“一锅法”则能使官能团直接参与到整体骨架中,官能团分布更均匀。

## 6 总结与展望

环境中元素形态分析依然面临着许多严峻的挑战,样品基质复杂、形态含量低、易转化成为形态分析中最棘手的难题,多形态同时分析将是元素形态分析的重要发展方向之一,同时,广泛种类的样品,如大气颗粒物、生物组织和体液中形态分析的任务将更加艰巨。基于SPE材料对形态进行分析前的样品预处理是一种有效应对以上难题的方法,但现有材料和方法还远不能满足相关研究的要求。因此,急需研究修饰多种功能基团、在不破坏原生形态的条件下上样且能在温和的SPE条件下选择性分离元素不同形态的分离材料。不难看出,基于“一锅法”RAFT反应,辅以DFT计算,制备方便、性能优良、操作简单的有机-无机杂化多功能离子印迹磁性纳米复合物是极佳的选择。

## References

[1] Templeton D M, Fujishiro H. Coordin Chem Rev, 2017, 352: 424

[2] Groenenbery J E, Lofts S. Environ Toxicol Chem, 2014, 33(10): 2181 24862928 10.1002/etc.2642

[3] Sun Y Y, Hu X, Wu J C, et al. Sci Total Environ, 2014, 493: 487 24964061 10.1016/j.scitotenv.2014.06.017

[4] Reid M S, Hoy K S, Schofield J R M, et al. TrAC-Trends Anal Chem, 2020, 123: 115770

[5] Rekhi H, Rani S, Sharma N, et al. Crit Rev Anal Chem, 2017, 47: 524 28644042 10.1080/10408347.2017.1343659

[6] Cheng H, Chen X, Shen L, et al. J Chromatogr A, 2018, 1531: 104 29173958 10.1016/j.chroma.2017.11.029

[7] Marcinkowska M, Baralkiewicz D. Talanta, 2016, 161: 177 27769396 10.1016/j.talanta.2016.08.034

[8] Li J, Liu J, Lu W, et al. Electrophoresis, 2018, 39: 1763

[9] Yang Y, Tan Q, Lin Y, et al. Anal Chem, 2018, 90: 11996 30182709 10.1021/acs.analchem.8b02607

[10] Clough R, Harrington C F, Hill S J, et al. J Anal At Spectrom, 2020, 35: 1236

[11] Milačič R, Ščančar J. TrAC-Trends Anal Chem, 2020, 127: 115888

[12] Wang H, Liu X, Nan K, et al. J Anal At Spectrom, 2017, 32: 58

[13] Viana J L M, Menegário A A, Fostier A H. Talanta, 2021, 226: 122119 33676674 10.1016/j.talanta.2021.122119

[14] Hu B, He M, Chen B B, et al. Spectrochim Acta B, 2013, 86: 14

[15] Okenicová L, Žemberyová M, Procházková S. Environ Chem Lett, 2015, 14(1): 67

[16] He M, Huang L, Zhao B, et al. Anal Chim Acta, 2017, 1: 973 10.1016/j.aca.2017.03.047 28502423

[17] Bendicho C, Bendicho-Lavilla C, Lavilla I. TrAC-Trends Anal Chem, 2016, 77: 109

[18] Jiang G B, et al. Environmental Sample Preparation. 2nd ed. Beijing: Chemical Industry Press, 2016

[19] Ma W B, Du X, Liu M M, et al. Chem Eng J, 2021, 412: 128576

[20] Karadjova I, Dakova I, Yordanova T, et al. J Anal At Spectrom, 2016, 31: 1949

[21] Xi Y, Shi H, Liu R, et al. J Hazard Mater, 2021, 416: 125772 33831704 10.1016/j.jhazmat.2021.125772

[22] Kong Z Y, Du Y J, Wei J F, et al. J Colloid Interface Sci, 2020, 588: 749 33317852 10.1016/j.jcis.2020.11.107

[23] Corps Ricardo A I, Abujaber F, Guzmán Bernardo F J, et al. TrAC-Trends Anal Chem, 2020, 27: e00097

[24] Faisal F, Qiao J Q, Lian H Z, et al. Talanta, 2020, 237: 122939 10.1016/j.talanta.2021.122939 34736670

[25] Huang Y, Wang R. Curr Org Chem, 2018, 22: 1600

[26] Fu J, Chen L, Li J, et al. J Mater Chem A, 2015, 3: 13598

[27] Branger C, Meouche W, Margaillan A. React Funct Polym, 2013, 73(6): 859

[28] Bayramoglu G, Arica M Y. J Hazard Mater, 2011, 187(1-3): 213 21272995 10.1016/j.jhazmat.2011.01.022

[29] Taghizadeh M, Hassanpour S. Polymer, 2017, 132: 1

[30] Liang Q, Geng J, Luo H, et al. J Mol Liq, 2017, 248: 767

[31] Huang R, Ma X, Li X, et al. J Colloid Interface Sci, 2018, 514: 544 29291553 10.1016/j.jcis.2017.12.065

[32] Kong D, Qiao N, Wang N, et al. Phys Chem Chem Phys, 2018, 20(18): 12870 29700530 10.1039/c8cp01163j

[33] Elsayed N H, Monier M, Alatawi R A S, et al. Carbohydr Polym, 2022, 284: 119139 35287888 10.1016/j.carbpol.2022.119139

[34] Mesa R L M, Villa J E L, Khan S, et al. Nanomaterials, 2020, 10(12): 2541 10.3390/nano10122541 PMC7766906 33348754

[35] Cui Y, Liu J Q, Hu Z J, et al. Anal Methods, 2012, 4(10): 3095

[36] Tarley C R T, Andrade F N, de Oliveira F M, et al. Anal Chim Acta, 2011, 703(2): 145 21889628 10.1016/j.aca.2011.07.029

[37] Mishra S, Verma N. Chem Eng J, 2017, 313: 1142

[38] Kisomi A S, Khorrami A R, Alizadeh T, et al. Ultrason Sonochem, 2018, 44: 129 29680594 10.1016/j.ultsonch.2018.02.019

[39] Birlik E, Ersöz A, Açıkkalp E, et al. J Hazard Mater, 2007, 140(1/2): 110 17074437 10.1016/j.jhazmat.2006.06.141

[40] Gallego-Gallegos M, Muñoz-Olivas R, Cámara C, et al. Analyst, 2006, 131(1): 98 16365669 10.1039/b511946d

[41] Cai X, Li J, Zhang Z, et al. ACS Appl Mater Interfaces, 2014, 6(1): 305 24344795 10.1021/am4042405

[42] Abdullah, Alveroglu E, Balouch A, et al. J Polym Res, 2020, 27(9): 261

[43] Liu Y, Meng X, Han J, et al. J Sep Sci, 2013, 36(24): 3949 24151162 10.1002/jssc.201300972

[44] Fei J J, Wu X H, Sun Y L, et al. Anal Chim Acta, 2021, 1162: 338477 33926696 10.1016/j.aca.2021.338477

[45] Fan H, Fan X, Li J, et al. Ind Eng Chem Res, 2012, 51: 5216

[46] Xu S, Chen L, Li J, et al. J Hazard Mater, 2012, 237: 347 22981287 10.1016/j.jhazmat.2012.08.058

[47] Zhao B S, He M, Chen B B, et al. Microchim Acta, 2019, 186(12): 775 10.1007/s00604-019-3819-5 31728641

[48] Yang X, Zhang Z H, Zhang H B, et al. Chinese Journal of Analytical Chemistry, 2011, 39(1): 5

[49] Gómez-Romero P, Sanches C. Functional Hybrid Materials. 1st ed. Weinheim: Wiley-VCH, 2004

[50] Zheng F, Hu B. Spectrochim Acta B, 2008, 63(1): 9

[51] Zhang Z H, Zhang M L, Xu T Z, et al. Chemical Journal of Chinese Universities, 2010, 31(9): 1734

[52] Liu Y, Meng X G, Liu Z C, et al. Langmuir, 2015, 31: 8841 26204060 10.1021/acs.langmuir.5b01201

[53] Zhang M Y, Ma X G, Huang R F, et al. Acta Scientiae Circumstantiae, 2019, 39(9): 8

[54] Wu G, Wang Z, Jie W, et al. Anal Chim Acta, 2007, 582(2): 304 17386507 10.1016/j.aca.2006.09.034

[55] Chiefari J, Chong Y K, Ercole F, et al. Macromolecules, 1998, 31(16): 5559

[56] Meng M, Meng X, Liu Y, et al. J Hazard Mater, 2014, 278: 134 24956578 10.1016/j.jhazmat.2014.06.002

[57] Liu Y, Qiu J, Jiang Y, et al. Micropor Mesopor Mater, 2016, 234: 176

[58] Zhao L Y, Fei J J, Lian H Z, et al. Talanta, 2020, 212: 120799 32113561 10.1016/j.talanta.2020.120799

[59] Zhao L Y, Zhu Q Y, Mao L, et al. Talanta, 2019, 192: 339 30348400 10.1016/j.talanta.2018.09.064

[60] Li P, Zhang X Q, Chen Y J, et al. RSC Adv, 2014, 4(90): 49421

[61] Boyacı E, Çağır A, Shahwan T, et al. Talanta, 2011, 85:1517 21807217 10.1016/j.talanta.2011.06.021

[62] Zheng N J, Tan J H, Duan J C, et al. Environmental Chemistry, 2014, 33(12): 2109

[63] Zhong L J, Tang Z J, Hu X, et al. Progress in Chemistry, 2021, 33(10): 1766

[64] Tavengwa N T, Cukrowska E, Chimuka L. Talanta, 2013, 116: 670 24148460 10.1016/j.talanta.2013.07.034

[65] Xiao H M, Cai L, Zhang Z H, et al. Chinese Journal of Applied Chemistry, 2020, 37(9): 11

[66] Hua Y, Zhang S, Min H, et al. At Spectrosc, 2021, 42(4): 217

[67] Hua Y, Li J Y, Min H, et al. Microchem J, 2020, 158: 105210

